# Mineralogy and heavy metal assessment of the Pietra del Pertusillo reservoir sediments (Southern Italy)

**DOI:** 10.1007/s11356-020-10829-6

**Published:** 2020-09-19

**Authors:** Roberto Buccione, Elisabetta Fortunato, Michele Paternoster, Giovanna Rizzo, Rosa Sinisi, Vito Summa, Giovanni Mongelli

**Affiliations:** 1 Department of Sciences, University of Basilicata, viale dell’Ateneo Lucano 10, 8500 Potenza, Italy; 2 Istituto Nazionale di Geofisica e Vulcanologia, via Ugo La Malfa, 153 90156 Palermo, Italy; 3 National Research Council-Institute of Methodologies for Environment Analysis, C.da S. Loja-Zona Industriale, 85050 Tito Scalo, PZ Italy

**Keywords:** Heavy metals, Mineralogy, Lake sediments, Environmental quality, Risk assessment, Enrichment factors

## Abstract

The Pietra del Pertusillo freshwater reservoir is a major artificial lake of environmental, biological, and ecological importance located in the Basilicata region, southern Italy. The reservoir arch-gravity dam was completed in 1963 for producing hydroelectric energy and providing water for human use, and nearby there are potential sources of anthropogenic pollution such as urban and industrial activities. For the first time, the minero-chemistry of the lake and fluvio-lacustrine sediments of the reservoir have been evaluated to assess the environmental quality. Moreover, the composition of fluvial sediments derived from the peri-lacual zone of the reservoir and of local outcropping bedrock were also studied to understand the factors affecting the behavior of elements in the freshwater reservoir, with particular attention paid to heavy metals. In Italy, specific regulatory values concerning the element threshold concentration for lake and river sediments do not exist, and for this reason, soil threshold values are considered the standard for sediments of internal waters. The evaluation of the environmental quality of reservoir sediments has been performed using enrichment factors obtained with respect to the average composition of a reconstructed local upper continental crust. We suggest this method as an innovative standard in similar conditions worldwide. In the studied reservoir sediments, the trace elements that may be of some environmental concern are Cr, Cu, Zn, As, and Pb although, at this stage, the distribution of these elements appears to be mostly driven by geogenic processes. However, within the frame of the assessment and the preservation of the quality of aquatic environments, particular attention has to be paid to As (which shows median value of 10 ppm, reaching a maximum value of 26 ppm in Quaternary sediments), constantly enriched in the lacustrine samples and especially in the fine-grained fraction (median = 8.5 ppm).

## Introduction

Freshwater reservoirs role great importance for investigating pollution as they are generally located close to the urban and industrial sources of pollution, where contamination issues are most likely to arise (Ammar et al. 2015; Baran et al. 2016). The reservoir sediments can be sensitive indicators for monitoring contaminants in aquatic environments (Liu et al. 2018; Aung et al. 2019; Kulbat and Sokołowska 2019; Zhuang et al. 2019), acting as sinks for different pollutants. In particular, clay fraction is mainly enriched of heavy metals, especially in highly polluted sediments (Uddin 2017; Jones et al. 2019). Depending on the environmental conditions (sediment pH, temperature, and redox status) and on the texture and composition of the sediments heavy metals may be released and back into the water column, thus impacting the overlying water quality (Mongelli et al. 2015; Ammar et al. 2015). Furthermore, the deep reservoir sediments represent the historical memory of an ecosystem, recording the past environmental changes caused by both natural and artificial events; they allow us to reconstruct the history of depositional events and define their possible origin (Islam et al. 2015). Due to their rapid sedimentation rates, reservoir sediments are considered being little affected by early diagenesis processes and provide preserved historical heavy metal inputs (Audry et al. 2004; Xu et al. 2017; Zhang et al. 2017).

Heavy and critical metals are among the most common environmental pollutants stressing the biotic community and are released into the environment by the weathering of rocks (e.g., Mongelli et al. 2014a) or by human activities, such as mining, industrial and domestic effluents, and combustion of fossil fuels. They may be transported by channelized flows (such as rivers and streams), or by atmospheric dust and accumulate in natural depressed areas (lakes, seas, and oceans). If it happens, metals may represent a significant part of sediment contaminants and, at some concentrations, they may become toxic to the environment (Farkas et al. 2007; Tuikka et al. 2011; Shyleshchandran et al. 2018; Christophoridis et al. 2020).

In aquatic systems, heavy metals can be dissolved into solutions as free-ions for a short time; they, in fact, commonly are suspended as colloids by adsorption onto inorganic compounds (such as iron-manganese oxides and hydroxides and clay minerals) and organic matter (the most reactive phases in aquatic environments) or precipitated with oxides and carbonates (De Vivo et al. 2004; Ammar et al. 2015). For this reason, concentration of heavy metals might serve as useful indicators for the appropriate assessment of sediment contamination.

Although the use of lacustrine sediments as environmental archives is well established, reservoir sediments have less frequently been used as temporal records and much less common studies using sediment cores from artificial river impoundments or reservoirs (Harikumar et al. 2009; Gao et al. 2018; Varol et al. 2020). Otherwise, what is known about sediment distribution in natural lakes may not apply to reservoirs because reservoirs have a component of riverine hydrodynamics that natural lakes tend to lack (Abraham et al. 1999). Abraham et al. (1999) provided useful knowledge on the processes and consequently on the factors affecting the distribution of sediments in reservoirs.

The PPR, characterized by a 95-m-height arch-gravity dam, was completed in 1963 for producing hydroelectric energy and providing water for human use to the Apulia and Basilicata regions. The reservoir has a water capacity of 155 million m^3^ and is characterized by strong seasonal fluctuations of the water level as high as 40 m, mainly due to seasonal rainfall/discharge variations.

Because of its great importance from the environmental, biological, and ecological points of view, the PPR has been the subject of several studies about the quality of its waters but poor importance has been given to its fluvial and lacustrine sediments. The available studies carried out on the sediments filling the PPR are discontinuous and fragmented and, in any case, have interested only the shallow portion of lacustrine sediments thus preventing to assess the factors affecting the lake sediments composition and understand the origin of potential pollutants.

With this in mind, we present a comprehensive and detailed minero-chemical characterization of the lacustrine sediments from the PPR based on (a) the assessment of the average chemical composition of the upper continental crust close to PPR, (b) the minero-chemical composition of the detrital supply close to the entry points of the reservoir, and (c) the distribution and the concentrations of trace elements in the bulk and fine fraction (< 4 μ) of deep lacustrine sediments. Moreover, the composition of fluvial sediments derived from the peri-lacual zone of the reservoir and of local outcropping bedrock (Meso-Cenozoic rocks and Quaternary fluvio-lacustrine deposits) were also studied. The goal of the present paper is to understand the environmental factors influencing the geochemical behavior of elements in an artificial freshwater reservoir which role a critical impact for a great number of people and environment health, with particular attention to heavy metals, providing a model useful in other similar scenarios worldwide.

## Material and methods

### Study area and geological setting

**Fig. 1:**
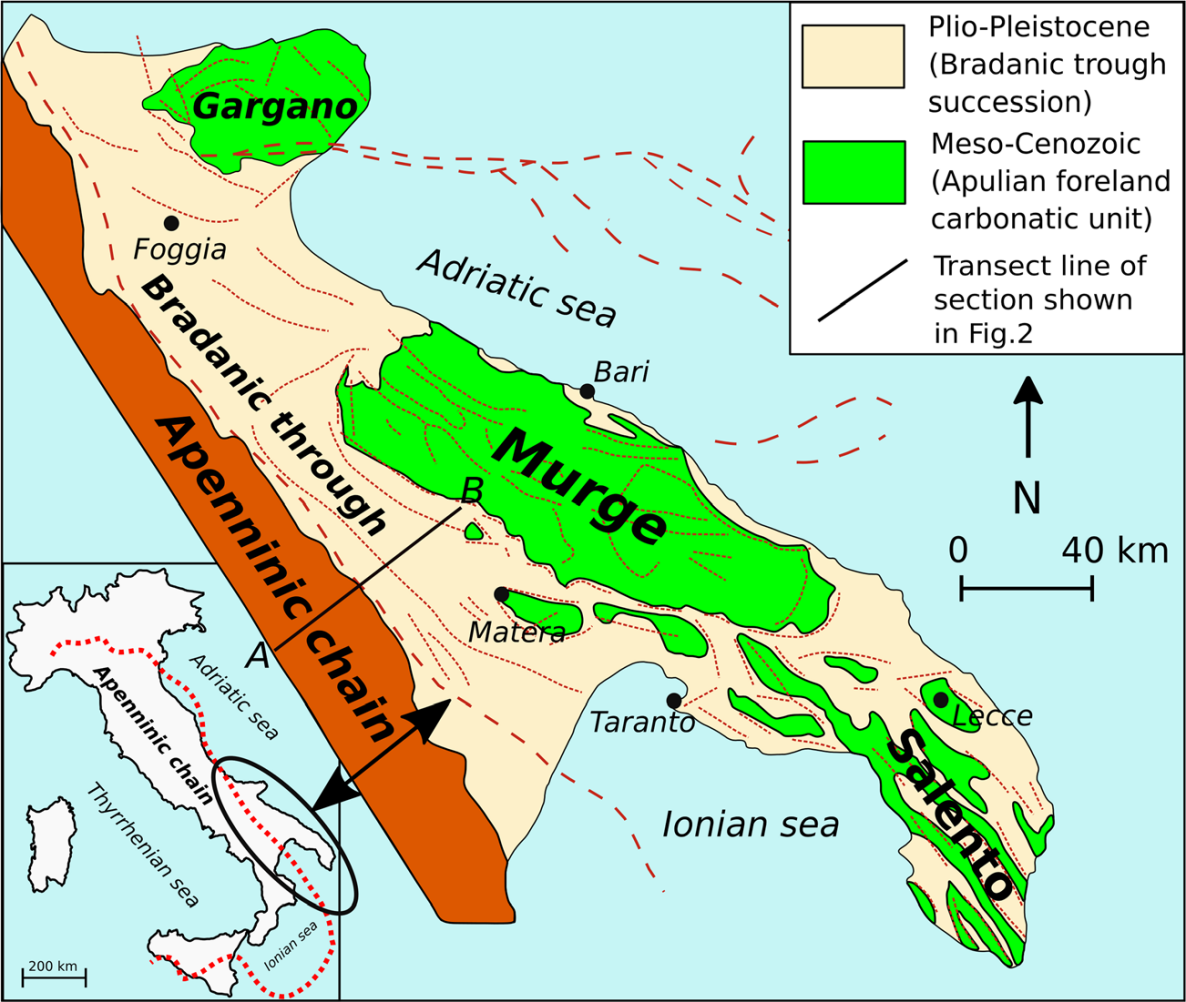
Geology of the three main paleogeographic domains of Southern Apennines (modified from Prosser et al. 1996)

The Pietra del Pertusillo reservoir (hereafter PPR) is an NW–SE-elongated artificial lake located in the southwestern Basilicata region (southern Italy). The reservoir is located in the High Agri Valley (hereafter HAV), which extends over a length of about 30 km and a maximum width of about 12 km, with an average altitude of 600 m a.s.l. and a surface of about 140 km^2^ (Fig. 1). It is mainly sourced by the Agri River, which enters longitudinally the lake basin from the West, whereas other minor lateral tributaries on both northern and southern margins, and during the high-stand periods, are also present, with a torrential-type discharge regime.

The total volume of sediments accumulated in the reservoir in the period 1963–2007 was about 7,420,000 m^3^ with the greatest sedimentation in the pre-reservoir Agri channel; here, the sediment thickness reaches a maximum value of about 8.7 m in the deepest area of the reservoir, near its dam, where the approximate average sedimentation rate reaches the maximum value of about 19.7 cm/yr.

**Fig. 2:**
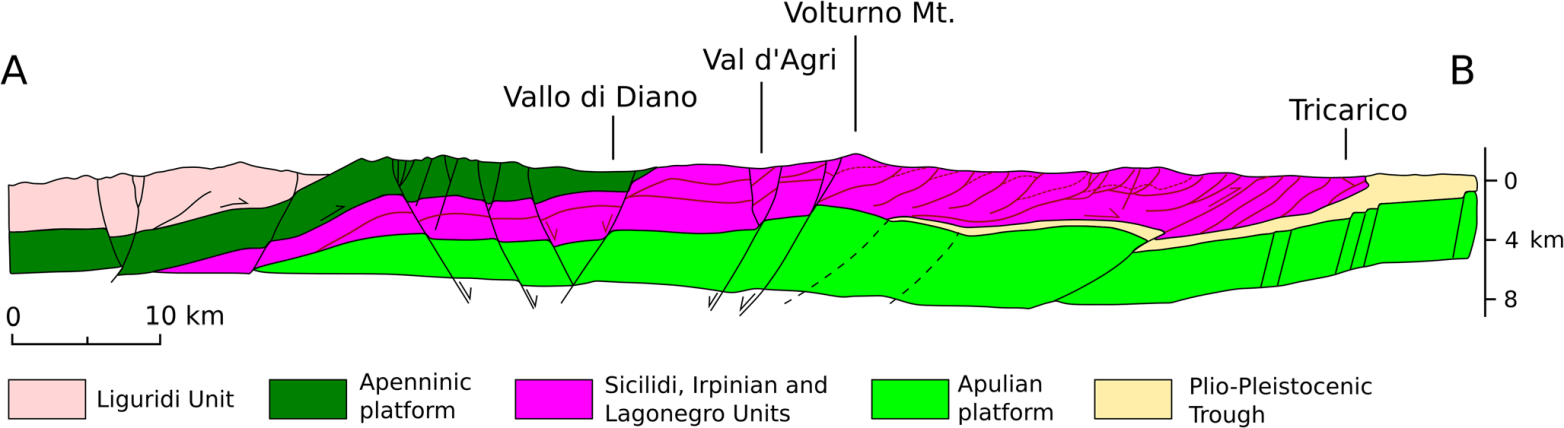
Relationships between the different tectonic units in the Southern Apennines (modified from Prosser et al. 1996)

The High Agri Valley is a Quaternary NW–SE intermontane basin located in the axial zone of the Southern Apennines mountain belt, an east-verging fold-and-thrust belt developed as an accretionary wedge from the late Oligocene to the early Pleistocene, due to the eastward migration of the Apenninic arc (Fig. 1) (e.g., Giano 2011; Giocoli et al. 2015; Gueguen et al. 2015). The tectonic units, derived from the deformation of the Afro-Adriatic paleomargin, are represented from west to east by the Liguride and Sicilide complexes (Cretaceous to Eocene), derived from the Liguria-Piedmont Ocean; the Apennine Platform carbonates (late Triassic to Tertiary) and the several units mainly composed of deep-sea sediments of the Lagonegro basin (late Paleozoic/Triassic to Tertiary) (Zembo 2010; Giano 2011; Gueguen et al. 2015). These units (Fig. 2), representing the substratum of the HAV, were tectonically emplaced over Plio-Pleistocene foredeep basins located on top of the Apulian Carbonate Platform.

Quaternary tectonics is still active along the axis of the chain where normal faulting displaces the Pleistocene sediments. Toward the east and southeast, the bedrock consists of Tertiary siliciclastic sediments, namely, Albidona and Gorgoglione Flysch formations. The first formation (Albidona Flysch) is composed by an alternating gray-yellowish sandstone, marl, and silty clays with whitish carbonate intercalations outcropping in the eastern portion of the HAV basin. The Gorgoglione Flysch formed in a piggyback trough during the Apenninic orogenic compressional stage. This formation is mainly composed of three terrigenous sediment sequences outcropping in the eastern portion of the HAV basin (Carbone et al. 1991; Giano 2011). Continental clastic Quaternary units also crop out on both the right and left sides of the HAV, where about 100 m are exposed; it is a group of clastic units of mid-upper Pleistocene age, reflecting a progradation of fan systems in a lacustrine-palustrine setting, followed by expansion of a new alluvial-fluvial system (Zembo 2010).

### Sampling strategy

**Fig. 3:**
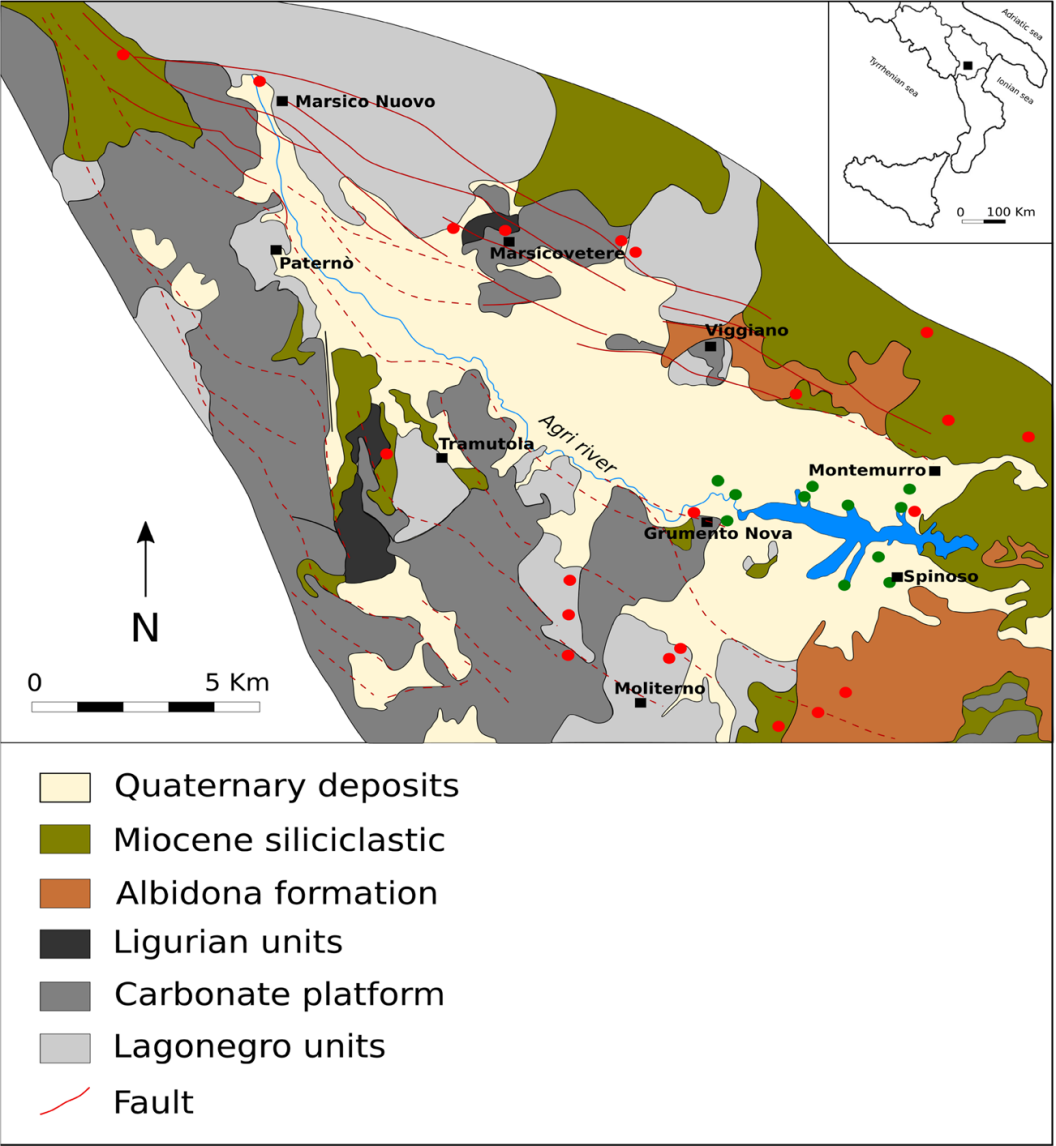
Sampling points of the twenty-seven Meso-Cenozoic lithoid samples

**Fig. 4:**
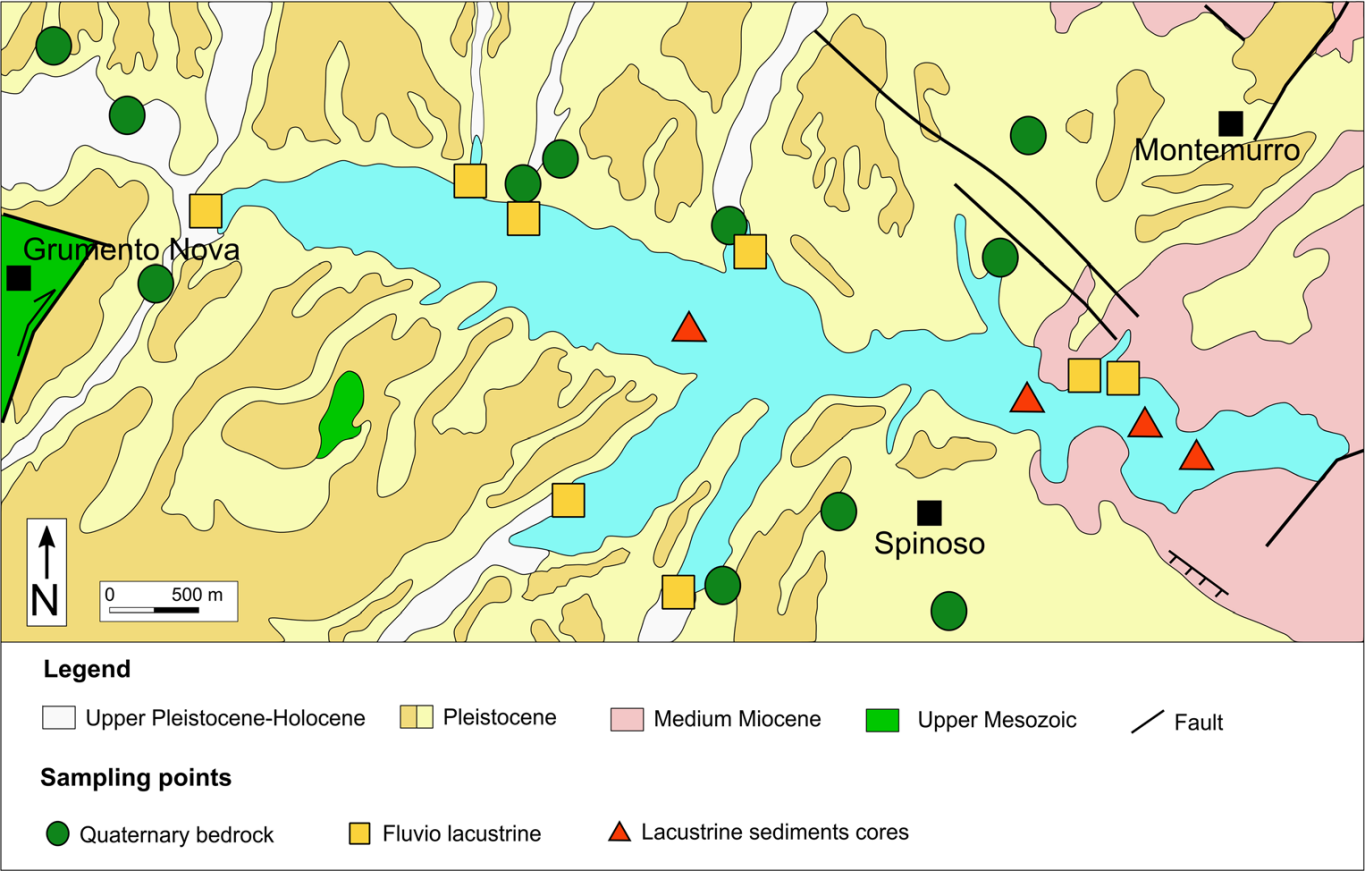
Samples position of quaternary bedrock deposits, fluvio-lacustrine sediments, and lake sediments cores (modified from Carbone et al. 1991)

The sampling campaign (Figs. 3 and 4) was performed in November 2013 and May 2014 and, with this in mind, several specimens were collected (Table 1) as follows:

- In the surrounding area of the reservoir, twenty-seven Meso-Cenozoic parent rock samples, with a predominate siliciclastic pelitic fraction
- Fifteen silty-clayey samples from the Quaternary fluvio-lacustrine deposits, outcropping in the drainage basin and, during the low-standing periods, along the shores of the reservoir
- Fourteen fluvio-lacustrine sediments samples. These samples were collected at the confluences of the active tributaries of the reservoir
- Fifteen samples from lake-bottom sediments, from 4 sediment cores (F, R, T, and V), lacustrine sediments were investigated, with a small platform that allowed to drill cores up to 2 m long from the sediment/water interface

**Table 1:** Collected samples. For Meso-Cenozoic lithoid samples, relative lithoid formation is reported as well

Lacustrine sediments (bulk fr.)	Lacustrine sediments (clay fr.)	Fluvio-lacustrine sediments	Meso-Cenozoic deposits	Quaternary incoherents sediments
F1 3	F1 3	EFF1	*Moliterno fm.*	EF39
F1 6	F1 6	EFF2	EF1	EF41
F1 12	F1 12	EFF3	EF5	EF49
a/R	a/R	EFF4	EF6	EF50
R9	R9	EFF5	EF81	EF51
R12	R12	EFF6	EF82	EF52
R18	R18	EFF7	*Albidona fm.*	EF54
T1	T1	EFF8	EF16	EF60
a/T	a/T	EFF9	EF79	EF61
c/T	c/T	EFF10	EF84	EF62
a/V	a/V	EFF11	EF57	EF63
b/V	b/V	EFF12	EF91	EF65
c/V	c/V	EFF13	*Gorgoglione fm.*	EF67
d/V	d/V	EFF14	EF22	EF71
V3	V3	–	EF26	EF74
–	–	–	EF83	–
–	–	–	EF78	–
–	–	–	EF 59	–
–	–	–	*Galestri fm.*	–
–	–	–	EF28	–
–	–	–	EF30	–
–	–	–	EF76	–
–	–	–	EF77	–
–	–	–	EF88	–
–	–	–	EF89	–
–	–	–	*Scisti silicei fm.*	–
–	–	–	EF80	–
–	–	–	EF85	–
–	–	–	EF90	–
–	–	–	Liguridi	–
–	–	–	EF86	–
–	–	–	EF87	–
–	–	–	*Monte Facito fm.*	–
–	–	–	EF34	–

### Mineralogical and chemical analyses

The Quaternary and fluvio-lacustrine sediments were wetly sieved, using a stainless steel ASTM 2-mm mesh sieve to separate the gravel from the sand and an ASTM 63 μm sieve to separate the sand from the silt-clay fraction. The sandy samples were dried at 105 °C, while the silt and clay fractions were preserved at room temperature to avoid deconstruction of the clays. Each sample was quartered and then a proper aliquot was hand-pulverized in an agate mill jar.

The selected lacustrine samples were wet sieved with stainless steel ASTM 63 μm sieve; the fraction < 4 μm was obtained by fractionated sedimentation. According to Stokes’ law, each sample after mechanical agitation of 8 h, was left to settle for a time of 1 h 57 min (the time calculated for a temperature of 22 °C which is the temperature recorded in the laboratory during the extraction procedures) from a drop height of 10 cm; once this time lapsed, the suspension was removed by siphoning. Extraction was repeated until the complete removal of the suspended solid.

**Fig. 5:**
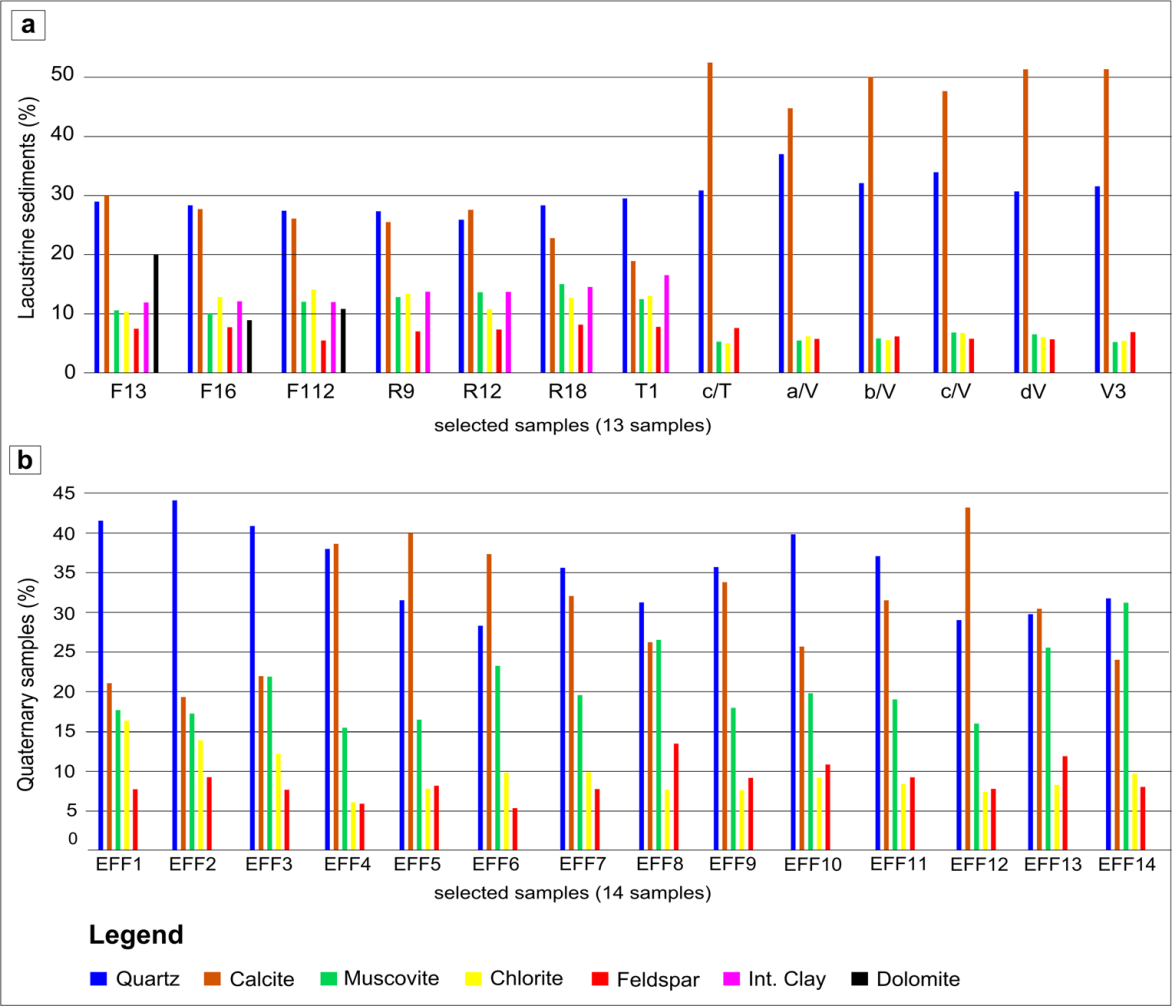
(a) Mineralogy composition of lake sediments. (b) Mineralogy composition of fluvio-lacustrine samples

Mineralogical analyses (Fig. 5) were performed on Quaternary fluvio-lacustrine samples related to the main tributaries of the reservoir and on the bottom lake lacustrine sediments.

Semiquantitative mineralogical analysis of the lacustrine and fluvio-lacustrine sediments (fraction lower than 63 μm) was determined by X-ray diffraction (X’Pert PRO Panalitycal diffractometer for powders) on non-oriented powders (random powder), obtained with the “top-loading” method of the sample holder. The X-ray diffraction analysis of the fluvio-lacustrine sediments (14 samples) was carried out at a scan interval of 2–70° 2ϑ, using acquisition steps of 0.02° 2ϑ; on the lacustrine sediments (15 samples), the scan interval was 5–35° 2ϑ, using acquisition steps of 0.01° 2ϑ, time per step = 1 s; copper radiation Cu-Kα1,2 = 1.54184 was used.

Powdered samples were digested using a four acid attack (HF, HNO_3_, HClO_4_, and HCl). A 0.25-g sample is first digested using hydrofluoric acid; then, the sample is treated in a mixture of nitric and perchloric acids before being heated in several ramping and holding cycles, taking the samples to incipient dryness. Next, the samples are brought back into solution using aqua regia before being analyzed. Major and trace elements were determined by inductively coupled plasma optical mass spectrometry (ICP-MS), using a PerkinElmer Sciex ELAN 9000 at Activation Laboratories (Ancaster, Canada).

Analytical data quality was checked by using NIST694 and DNC-1(for major element), JR-1 (for As and Pb), and GXR-4 (for Cr, Ni, and V) Standard Reference Materials (SRM) and by calculating the average relative error. SRM of known elemental composition play an important role in the quality assurance (QA). For most elements, the recovery quality assurance/quality control (QA/QC) ranges between 80 and 110%. Analytical uncertainties are less than ±5%, except for elements at concentrations of ≤ 10 ppm which have uncertainties of ±5–10%. Total loss-on-ignition (LOI) values was gravimetrically estimated after overnight heating at 950 °C. Total organic carbon (TOC) was evaluated, by loss-on-ignition, only for lacustrine sediments. Three grams of wet sediment was placed in a drying oven at 60 °C for 48 h then cooled in a desiccator and weighted, subsequently was placed in a muffle furnace at 500 °C for 8 h, and finally cooled in a desiccator and weighted for the no-organic matter dried sediment (wt_60°C_). After a week, the samples were re-introduced in the muffle furnace at 500 °C for 8 h and then reweighted (wt_500°C_).

The percentage of organic matter was calculated by the following formula (Hakanson and Jansson 1983):

$$ \mathrm{TOC}=\left({\mathrm{wt}}_{60{}^{\circ}\mathrm{C}}-{\mathrm{wt}}_{500{}^{\circ}\mathrm{C}}\right)/\left({\mathrm{wt}}_{60{}^{\circ}\mathrm{C}}\right)\times 100, $$

where wt_60°C_ and wt_500°C_ are the weight of sample after heating at 60 °C for 48 h and at 500 °C for 8 h, respectively.

### Statistical data treatment

**Fig. 6:**
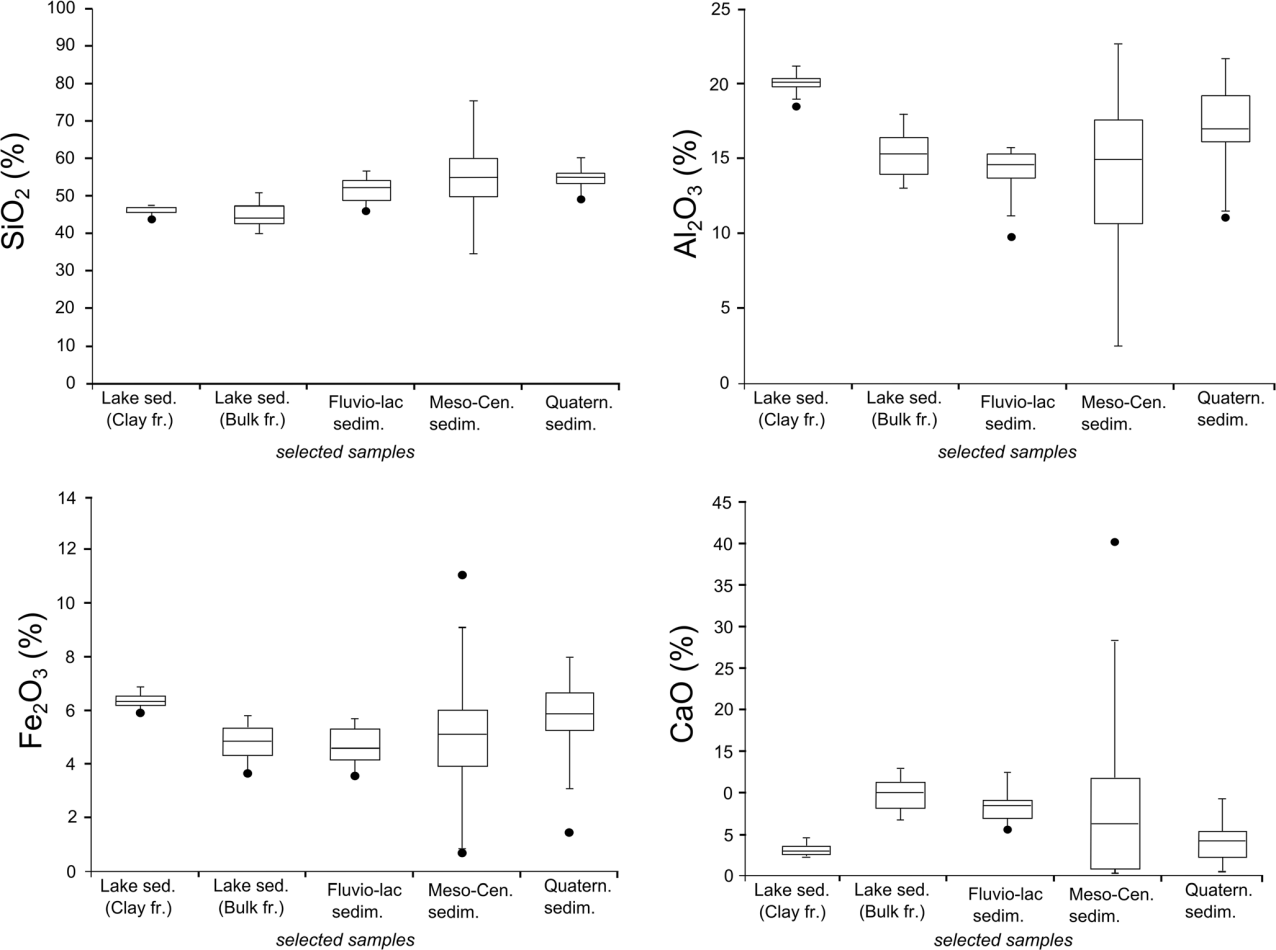
Box-and-whisker plots for major elements in the analyzed samples. The horizontal bar in the box refers to the median value; the ends of the whiskers are the maximum and minimum values of variables; the top and bottom of the boxes are the values of first and third quartiles; dark circles represent the outliers values of the dataset

Used statistical data relative to the analyzed samples are reported in Fig. 6 with the box-and-whiskers plot.

In particular, among the statistical data, for each set of sample’s minimum value, maximum value, and the relative range (difference between maximum and minimum values) were calculated. Then, the 25th, 50th (median), and 75th percentile of each data set have been calculated. In relation to the latter data, interquartile range (IQR) (being equal to the difference between 75th and 25th percentiles or between upper and lower quartiles) has been obtained. IQR is a measure of variability, based on dividing a data set into quartiles. Finally, outlier values (data point that differs significantly from other values) has been calculated and reported into the box-and-whiskers plot.

### Enrichment factors

In order to evaluate the possible contamination of the analyzed samples, the enrichment factors (EFs) for metals of environmental interest were calculated.

In the present study, the EFs were calculated using the UCC (upper continental crust, McLennan et al. 2006) and local bedrock compositions as reference standards. The used equations are as follows (Reimann and De Caritat 2005):

(i) EF respect to UCC = (*X*/Ti)_sample_/(*X*/Ti)_UCC_
(ii) EF respect to local bedrock = (*X*/Ti)_sample_/(*X*/Ti)_loc.bed._

where *X* and Ti are the weight percentages of a given element and titanium, respectively.

Titanium was used as a normalizing element because it is associated with crustal rock sources and it is characterized by negligible mobility during weathering and diagenesis (e.g., Mongelli et al. 2014b and references therein). Five categories for the EF index are recognized as follows (Sutherland 2000): EF < 2 is depletion to minimal enrichment, EF 2–5 is moderate enrichment, EF 5–20 is significant enrichment, and EF 20–40 is extremely high enrichment.

## Results

### Mineralogy

Results of mineralogical analyses are presented in Table 2.

**Table 2:** Mineralogical composition (in percentage) of lake and fluvio-lacustrine samples

	Quartz (%)	Calcite (%)	Feldspar (%)	Muscovite/illite (%)	Chlorite (%)	Interstr. clay (%)	Dolomite (%)
*Lake sediments*							
F1 3	23.5	23.5	7.0	10.5	4.7	11.1	19.5
F1 6	25.4	27.5	4.7	10.3	10.7	12.0	9.2
F1 12	27.2	20.7	5.4	10.5	13.9	11.4	10.6
R9	27.3	25.5	7.1	12.5	13.4	14.0	nd
R12	26.2	27.4	7.5	13.7	10.9	14.0	nd
R18	28.1	22.3	7.9	14.6	12.3	14.4	nd
T1	29.5	19.0	8.2	12.7	13.5	16.9	nd
c/T	30.4	52.2	7.3	5.2	4.7	nd	nd
a/V	37.2	44.8	6.0	5.6	6.2	nd	nd
b/V	32.2	50.0	6.2	5.9	5.5	nd	nd
c/V	33.9	47.2	5.5	6.8	6.3	nd	nd
d/V	30.6	51.1	5.6	6.5	6.1	nd	nd
V3	31.4	51.0	7.0	5.0	5.3	nd	nd
*Fluvio-lacustrine sedim.*							
EFF1	40.0	20.2	7.1	16.8	15.6	nd	nd
EFF2	42.8	18.5	8.8	16.5	13.1	nd	nd
EFF3	39.3	21.1	7.2	20.7	11.5	nd	nd
EFF4	36.5	36.9	5.7	14.8	5.8	nd	nd
EFF5	30.3	38.1	7.9	15.8	7.6	nd	nd
EFF6	27.4	35.8	5.2	21.7	9.7	nd	nd
EFF7	34.1	30.8	6.9	18.8	9.2	nd	nd
EFF8	30.0	24.9	12.6	25.2	7.1	nd	nd
EFF9	34.4	32.5	8.8	17.2	7.0	nd	nd
EFF10	37.9	24.5	10.0	18.8	8.7	nd	nd
EFF11	35.4	30.1	8.4	17.9	8.0	nd	nd
EFF12	27.5	42.7	7.3	15.2	7.0	nd	nd
EFF13	28.2	28.4	11.2	24.2	7.7	nd	nd
EFF14	30.4	22.8	7.5	29.5	9.5	nd	nd

nd: not detected

Mineralogical phases were determined based on relative intensity of higher peaks. The most abundant mineralogical phases detected in the analyzed samples are mainly quartz and calcite. The other mineralogical phases are feldspars, muscovite/illite, interstratified clay minerals, and chlorite. Dolomite is present only in three fluvio-lacustrine samples.

### Fluvio-lacustrine samples

In the quaternary fluvio-lacustrine deposits, quartz reaches maximum values in sample EFF2 (42.80%) and shows a median of 34.29%. Calcite ranges from 18.59 to 42.78% with a median of 29.31%. Feldspars (median = 7.76%) and chlorite (median = 8.38%) show quite similar abundances in all fluvio-lacustrine samples. Finally, muscovite/illite median is 18.40% with a range from 14.84 to 29.59%.

The relative abundance of quartz and calcite, which are the main mineralogical phases, is variable for lacustrine samples (Fig. 5a, b). In the F core, quartz shows a median value of 25.38% while calcite has a median of 23.92%. In the R core, quartz (median = 27.25%) and calcite (median = 25.2%) show very similar values. In the T core, quartz has a median of 29.98% while calcite shows a median of 35.66%. In the V core, calcite abundance is higher with respect to quartz with median values of 48.85% and 33.11%, respectively.

### Lake sediments

For all lake samples, feldspars display quite similar values with a median of 7.05%, a minimum of 4.73% and a maximum of 8.21%.

Muscovite/illite present a median values of 10.23% ranging from 5.04 to 14.69%, and they show maximum values in core R (max = 13.74%) and minimum value in core V (5.04%).

Chlorite (median = 6.39%, min = 4.70, max = 13.96%) is higher in core F (median = 10.75), R (median = 12.36), and T (median = 9.10%) while is less abundant in core V (median = 6.11).

Interstratified clay minerals are present only in cores F, R, and T, and this mineralogical phase presents a median of 14.05%, maximum value of 16.95%, and a minimum of 9.21%.

Dolomite is present in the core F only showing a minimum of 9.21%, a maximum of 19.50%, and a median of 10.63%.

### Geochemistry

The most abundant major oxides are SiO_2_ and Al_2_O_3_ usually followed by Fe_2_O_3_ and CaO (Table 3, Fig.6).

**Table 3:** Major oxides, selected trace elements, and REE values in the collected samples. The median value is displayed

	SiO_2_	Al_2_O_3_	Fe_2_O_3_	CaO	V	Pb	Cr	Co	Ni	Cu	Zn	As	∑REE
Units	%	%	%	%	ppm	ppm	ppm	ppm	ppm	ppm	ppm	ppm	ppm
d.l.	0.01	0.01	0.01	0.01	5	5	20	1	20	10	30	5	
*Lacustrine sediments (clay fraction)*													
F1 3	45.7	18.5	6.5	3.6	144	42	110	15	50	50	160	10	222
F1 6	43.8	19.7	6.4	4.4	148	44	120	15	50	50	150	8	221
F1 12	47.5	21.3	7.4	2.4	164	46	130	20	60	60	140	8	255
a/R	46.9	19.8	7.1	2.0	154	43	120	17	60	70	220	10	298
R9	45.8	20.2	6.8	2.7	162	43	130	15	50	50	150	8	231
R12	45.3	20.0	7.1	2.9	159	43	130	15	50	60	160	8	219
R18	46.7	20.0	6.9	3.5	158	35	130	15	50	50	150	< 5	235
T1	45.8	20.5	6.9	2.5	157	38	130	16	50	50	140	7	243
a/T	44.5	19.2	6.7	3.6	145	34	120	16	50	50	130	8	220
c/T	46.7	19.7	6.8	2.4	159	36	120	18	50	50	140	10	236
a/V	47.3	20.5	7.0	2.4	159	49	120	16	50	50	130	8	244
b/V	46.1	19.7	6.7	2.4	151	43	120	17	50	50	160	9	230
c/V	45.9	20.0	6.8	2.4	152	31	120	17	50	50	130	10	237
d/V	47.1	18.3	6.6	3.1	147	33	120	17	50	50	130	10	209
V3	46.6	20.3	7.1	2.8	153	42	120	16	50	50	150	9	241
*Median*	46.1	20.0	6.8	2.7	154	42	120	16	50	50	150	8	235
*Lacustrine sediments (bulk fraction)*													
F1 3-B	47.5	12.9	4.5	9.8	91	22	80	13	30	40	100	< 5	172
F1 6-B	41.6	13.8	4.8	12.7	105	23	90	13	40	40	110	< 5	179
F1 12-B	50.2	14.1	4.9	8.1	102	25	90	15	40	40	100	< 5	185
a/R-B	42.6	14.7	5.2	12.1	109	22	90	13	40	40	120	< 5	181
R9-B	50.8	13.4	4.4	9.2	93	24	80	10	40	30	100	< 5	180
R12-B	50.8	12.9	4.1	10.0	81	26	80	10	30	30	110	< 5	170
R18-B	47.1	15.2	5.3	9.1	114	27	100	13	40	40	120	< 5	199
T1-B	44.1	17.9	6.2	6.5	130	35	110	14	50	50	130	5	217
a/T-B	42.6	15.5	5.5	10.4	120	21	90	13	40	40	110	9	189
c/T-B	39.8	15.4	5.6	12.0	122	21	100	15	40	40	100	8	177
a/V-B	43.6	17.7	6.3	7.5	137	27	110	15	50	50	140	7	218
b/V-B	41.0	15.4	5.5	10.8	119	22	100	14	40	40	110	6	184
c/V-B	44.4	17.4	6.1	7.7	135	25	110	14	40	50	120	7	207
d/V-B	43.5	13.8	5.0	11.5	107	22	100	12	50	40	100	< 5	169
V3-B	45.3	17.0	6.1	7.5	134	27	110	14	50	50	110	< 5	214
*Median*	44.1	15.2	5.3	9.8	114	24	100	13	40	40	110	7	184
*Fluvio-lacustrine sediments*													
EFF1	53.3	15.3	5.9	6.5	113	33	63	14	57	40	143	8	188
EFF2	54.2	15.2	5.8	6.0	113	25	78	13	59	30	140	7	194
EFF3	54.2	15.6	5.9	5.4	119	21	73	17	60	50	141	7	195
EFF4	53.4	12.7	4.4	8.6	92	22	59	11	43	30	106	7	183
EFF5	48.6	14.9	6.2	9.4	111	52	64	16	58	40	153	9	213
EFF6	47.0	15.4	5.5	9.6	117	22	102	13	49	30	133	6	187
EFF7B	48.0	15.2	5.0	9.0	114	18	62	12	48	40	141	5	196
EFF8	48.8	13.1	4.5	8.0	93	23	83	10	43	40	209	6	171
EFF9B	48.6	12.4	4.1	8.4	86	26	58	9	42	40	180	6	171
EFF10	56.5	14.1	4.6	5.5	102	30	55	10	47	30	147	6	188
EFF11	50.2	14.0	4.7	7.7	96	17	61	10	43	30	129	4	188
EFF12B	54.0	9.7		12.8	58	14	46	7	31	20	85	3	137
EFF13	51.0	14.3	5.1	8.8	103	23	63	12	45	30	129	4	174
EFF14	54.2	14.7	5.7	6.9	105	43	58	14	50	30	149	7	186
*Median*	52.1	14.5	5.1	8.2	104	23	62	12	47	30	141	6	188
*Meso-Cenozoic samples*													
Moliterno succession													
EF1	74.6	10.9	2.9	1.8	84	6	60	20	60	50	30	5	152
EF5	55.4	15.0	5.6	7.4	108	16	80	15	30	20	90	7	188
EF6	58.8	18.1	7.9	1.1	93	21	90	12	30	40	80	8	223
EF81	61.0	19.3	6.2	0.1	139	8	100	14	40	30	100	5	176
EF82	56.2	20.6	7.4	0.3	137	21	110	17	40	30	110	5	184
Albidona Flysch													
EF16	48.3	14.6	4.4	11.5	119	23	90	13	40	50	110	5	200
EF79	20.3	5.3	2.0	38.0	52	6	50	13	40	40	50	5	262
EF84	17.7	4.7	1.7	39.3	50	6	50	9	40	30	50	5	214
EF57	41.4	12.5	4.5	18.7	101	18	70	8	37	40	90	5	178
EF91	16.4	4.3	1.7	40.9	46	6	40	8	30	30	40	5	150
Gorgoglione Flysch													
EF22	55.0	14.4	5.2	6.6	108	25	50	12	20	20	100	8	164
EF26	51.1	16.9	5.4	6.4	137	24	100.	13	30	30	110	7	205
EF83	90.9	2.4	1.2	0.9	20	5	20	7	20	30	30	5	150
EF78	49.0	15.1	5.3	11.1	116	23	80	9	30	30	110	6	184
EF 59	56.7	16.9	6.4	11.6	125	17	110	13	53	30	100	5	85
Galestri fm.													
EF28	50.8	22.0	11.5	0.1	157	26	120	50	50	30	140	10	221
EF30	58.5	19.5	6.3	0.2	135	21	110	35	50	50	140	5	223
EF76	64.0	14.8	7.8	0.8	107	14	70	21	70	110	60	5	215
EF77	50.5	10.2	4.1	15.8	96	10	50	8	50	90	60	5	28
EF88	66.4	14.2	7.3	0.2	117	6	100	22	100	50	110	5	76
EF89	38.7	9.7	6.4	20.6	87	5	70	15	40	10	100	5	142
Scisti silicei													
EF80	53.1	22.5	7.8	1.4	168	22	110	20	40	40	120	18	157
EF85	75.0	9.0	6.0	0.1	59	11	60	8	40	30	50	5	241
EF90	69.4	12.3	5.6	0.2	74	8	60	18	80	60	70	5	150
Ligurian complex													
EF86	55.4	16.3	4.9	6.8	163	9	100	17	40	30	90	5	120
EF87	52.2	21.3	6.6	2.4	154	13	110	20	40	90	110	9	216
Monte Facito fm.													
EF34	54.4	15.9	6.4	6.1	115	15	80	16	40	20	100	5	71
*Quaternary sediments*													
EF 39	54.6	17.0	6.5	4.0	126	34	100	20	48	40	110	9	211
EF 41	55.7	16.5	6.1	5.1	127	64	90	19	49	40	120	9	204
EF 49	55.1	20.1	7.4	0.4	147	60	100	18	63	50	140	10	273
EF 50	62.3	16.8	6.2	0.3	122	43	60	18	35	20	90	15	307
EF 51	56.1	15.9	4.8	5.8	117	23	90	8	38	20	100	5	190
EF 52	69.1	10.9	1.9	5.1	51	19	40	4	17	10	40	5	189
EF 54	49.9	13.9	8.5	9.0	130	79	80	27	55	70	150	26	164
EF 60	54.9	14.2	4.9	7.4	107	23	80	15	44	40	100	5	176
EF 61	52.5	21.5	5.3	1.1	176	30	120	12	47	50	130	10	284
EF62	53.3	18.8	7.5	2.9	149	50	110	21	40	50	130	12	216
EF63	58.6	16.5	6.4	3.1	127	23	100	16	40	40	110	7	191
EF65	55.8	16.2	6.2	4.2	119	30	90	20	40	40	120	13	192
EF67	52.4	19.4	7.4	3.2	153	30	110	20	50	50	120	10	243
EF71	53.1	17.5	6.5	4.2	125	31	80	15	30	40	120	8	233
EF74	53.3	21.0	7.0	1.2	159	32	110	19	40	50	130	10	262
*median*	54.9	16.8	6.4	4.0	127	31	90	18	40	40	120	10	211

d.l.: detection limit

### Lake sediments (bulk fraction)

In the bulk fraction of lacustrine samples, SiO_2_ shows a median of 44.17% and values which ranges from 39.88 to 50.82%; Al_2_O_3_ ranges from 12.93 to 17.94% with a median of 15.26. Fe_2_O_3_ has median values of 5.39% (min = 4.18%, max = 6.34%) while CaO content (median = 9.84%) varies from a minimum of 6.58 to a maximum of 12.72%. TOC in lacustrine sediments shows quite similar values, with a median value of 6.62% ranging from a minimum of 4.82 (sample R9) to a maximum of 9.79% (sample c/t).

V (med = 114 ppm), Cr (med = 100 ppm), and Zn (median = 110 ppm) are the most abundant trace elements with the higher variability values. Cu (med = 50 ppm) and Ni (median = 40 ppm) show very similar median values and IQR values. Pb ranges from 21 to 35 ppm with a median value of 24 ppm. Minor abundances were observed for Co (median = 13 ppm) and As (median = 8 ppm) with very lower range values of 5 and 6 ppm respectively. La (median = 39.2 ppm), Ce (median = 78.5 ppm), and Nd (median = 32.6 ppm) display always the REE higher concentrations. (La/Yb)_REE_ median is 1.19 and it has not been observed fractionation between HREEs and LREEs.

### Lake sediments (clay fraction)

In the clay fraction of lacustrine samples, SiO_2_ has a median of 46.18% ranging from 43.81 to 47.53%; Al_2_O_3_ varies from 18.38 to 21.3% with a median of 20.01%. The median value of Fe_2_O_3_ is 6.87% (min = 6.47%; max = 7.42%) while CaO (median = 2.78%) ranges from 2.06 (min) to 4.4% (max).

Concerning trace elements, V (median = 154 ppm), Cr (median = 120 ppm), and Zn (median = 150) display higher concentrations in clay fraction (< 2 μ) of lacustrine sediment samples. Pb (median = 42 ppm), Cu (median = 40), and Ni (median = 50) show similar median. Lower element enrichment quantities were observed for Co and As which show median values respectively of 16 and 4 ppm (less abundant element) and the same lower range value (IQR = 5). As it has been observed for bulk fraction, La, Ce, and Nd are the most abundant REE elements and they show median values of 53.7 ppm, 105 ppm, and 53.2 ppm, respectively. Among all the analyzed samples, these sediments display a moderate HREE/LREE fractionation with a (La/Yb)_UCC_ median value of 1.38.

### Fluvio-lacustrine samples

In the fluvio-lacustrine samples, SiO_2_ has a minimum of 47.04% and a maximum 56.51% with a median of 52.19%. Al_2_O_3_ ranges from 9.7 to 15.65% (median of 14.53%) while Fe_2_O_3_ has a median value of 5.11% with a range from 2.91 to 6.22%. The median value of CaO is 8.2%, the minimum is 5.42% while the minimum is 12.87%.

As concern trace elements in fluvio-lacustrine samples, Zn (med = 141 ppm) is the most concentrated element followed by V (med = 104 ppm), Cr (median = 62 ppm), Ni (median = 47.6 ppm), Cu (median = 30 ppm), and Pb (median = 23 ppm). Lower concentrations and range values for Co (median = 12 ppm) and As (median = 6.85) were observed. Also for these samples La, Ce, and Nd are the most abundant REEs showing median values of 40.05 ppm, 77.95 ppm, and 34.3 ppm, respectively. The (La/Yb)_UCC_ ratio has a median of 1.03 which reflecting no HREE/LREE fractionation.

### Meso-Cenozoic sediments

In the twenty-seven Meso-Cenozoic lithoid samples, SiO_2_ shows a range from 16.42 (Albidona fm.) to 90.93% (Gorgoglione fm.) with a median of 55%. Among the different sampled formations, the abundances of SiO_2_ are the main oxide and its distribution in the different lithologies is as follows: Moliterno succession (median = 58.86%); Albidona fm. (median = 20.32%); Gorgoglione fm. (median = 55%), Galestri formation (median = 54.74%), and Scisti silicei fm. (median = 69.49%); and Liguride fm. (median = 53.84%).

The distribution of Al_2_O_3_ shows a minimum of 2.44 (Gorgoglione fm.) and a maximum of 22.55% (Scisti silicei fm.) with a median of 14.88%; Al_2_O_3_ in the different formations is distributed as follows: Moliterno fm. (median = 18.10%); Albidona fm. (median = 5.33%); Gorgoglione fm. (median = 15.12%), Galestri fm. (median = 14.56%), and Scisti silicei fm. (median = 12.34%); and Liguride unit (median = 18.89%).

Fe_2_O_3_ has a median values of 5.65% ranging from 1.2 (Gorgoglione flysch) to 11.58% (Galestri formation). For each sampled formation, the abundances of Fe_2_O_3_ are the following: Moliterno succession (median = 6.2%); Albidona Flysch (median = 2.03); Gorgoglione Flysch (median = 5.38%), Galestri formation (median = 6.9%), and Scisti silicei (median = 6.01%); and Liguride complex (median = 5.8%).

CaO ranges from 0.13 (Albidona fm.) to 40.9% (Albidona fm.) and shows a median value of 6.13%., and in the different formations, the abundances of CaO are as follows: Moliterno fm. (median = 1.16%); Albidona fm. (median = 38.08%); Gorgoglione fm. (median = 6.64%), Galestri fm. (median = 0.58%), and Scisti silicei fm. (median = 0.29%); and Liguride unit (median = 4.65%).

In the Meso-Cenozoic bedrock samples, V (median = 108 ppm), Cr (median = 80 ppm), and Zn (median = 100 ppm) are the most abundant trace elements, showing higher variability with respect to other elements. Cu (median = 41.11 ppm) and Ni (median = 43.73 ppm) display similar distribution and concentrations with the presence of two and one upper outliers, respectively. Lower concentrations and variability for Co (median = 5 ppm), As (median = 14 ppm) and Pb (median = 14 ppm) were observed. La (median = 37.8 ppm), Ce (median = 76.6 ppm), and Nd (median = 30.6 ppm) are the most abundant REEs. The median value of total REE (∑REE) is 178.86 ppm; the (La/Yb)_UCC_ ratio is 1.03 excluding a significant fractionation between HREEs and LREEs. Among these samples three Ce-positive anomalies and three negative Ce anomalies have been observed.

### Quaternary sediments

In the Quaternary silty-clayey samples, the range values of major oxides are 49.90 to 69.14% (SiO_2_), 10.98 to 21.59% (Al_2_O_3_), 1.98 to 8.54% (Fe_2_O_3_), and 0.3% to 9.06% (CaO) while the median values are 54.93%, 16.89%, 6.41%, and 4.01% respectively.

The most abundant trace elements are V (median = 127 ppm), Cr (median = 90 ppm), and Zn (median = 120 ppm) followed by Pb (median = 31 ppm), Cu (median = 40 ppm), and Ni (median = 40 ppm). Lower concentrations and variability are related to Co (med = 18 ppm) and As (median = 10 ppm). The most abundant REEs are La, Ce, and Nd with median values of 43.4 ppm, 90.8 ppm, and 36.7 ppm, respectively. ∑REE median is 211.87. The (La/Yb)_UCC_ ratio shows a median value of 1.00 thus excluding any HREE/LREE fractionation.

### Enrichment factors (EFs)

Enrichment factors were calculated, relatively to UCC (McLennan et al. 2006) and local bedrock, for all the studied samples (Table 4).

**Table 4:** Trace elements enrichment factors values relatively to global UCC and local bedrock for the analyzed samples

	V		Cr		Co		Ni		Cu		Zn		Pb		As	
	(EF)_bedrock_	(EF)_UCC_	(EF)_bedrock_	(EF)_UCC_	(EF)_bedrock_	(EF)_UCC_	(EF)_bedrock_	(EF)_UCC_	(EF)_bedrock_	(EF)_UCC_	(EF)_bedrock_	(EF)_UCC_	(EF)_bedrock_	(EF)_UCC_	(EF)_bedrock_	(EF)_UCC_
*Lake sediments (clay fraction)*																
F_1_3	0.93	0.71	0.92	0.80	0.61	0.64	0.79	0.57	1.39	1.33	1.56	1.17	1.71	1.08	4.62	2.22
F_1_6	0.88	0.79	0.92	0.87	0.56	0.62	0.73	0.73	1.28	1.29	1.35	1.25	1.65	1.09	3.41	2.15
F_1_12	0.88	0.70	0.90	0.79	0.68	0.65	0.78	0.67	1.38	1.17	1.13	1.03	1.56	1.08	3.07	1.95
a/R	0.95	0.84	0.96	0.89	0.66	0.63	0.90	0.75	1.85	1.32	2.05	1.39	1.67	1.07	4.41	2.20
R9	0.98	0.77	1.02	0.85	0.57	0.52	0.74	0.80	1.30	1.06	1.37	1.24	1.64	1.24	3.46	2.35
R12	0.95	0.68	1.00	0.86	0.56	0.53	0.73	0.61	1.53	1.07	1.44	1.39	1.62	1.37	3.41	2.39
R18	0.99	0.81	1.05	0.92	0.59	0.58	0.76	0.69	1.34	1.22	1.42	1.29	1.38	1.21	1.79	2.03
T1	0.95	0.86	1.02	0.94	0.61	0.58	0.74	0.81	1.30	1.42	1.28	1.30	1.45	1.46	3.03	2.36
a/T	0.97	0.97	1.03	0.93	0.67	0.66	0.81	0.78	1.42	1.38	1.30	1.33	1.42	1.06	3.80	5.16
c/T	1.00	0.99	0.98	1.04	0.71	0.76	0.77	0.79	1.35	1.38	1.33	1.22	1.43	1.07	4.50	4.61
a/V	0.93	0.92	0.90	0.95	0.59	0.63	0.71	0.82	1.25	1.44	1.15	1.42	1.80	1.14	3.34	3.36
d/V	0.95	0.95	0.97	1.02	0.67	0.70	0.76	0.77	1.34	1.36	1.51	1.32	1.70	1.10	4.03	3.40
b/V	0.97	0.94	0.98	0.99	0.68	0.61	0.77	0.68	1.36	1.49	1.24	1.26	1.24	1.10	4.53	3.48
c/V	1.01	0.90	1.06	1.08	0.74	0.63	0.84	1.02	1.47	1.43	1.35	1.26	1.43	1.16	4.91	2.39
V3	0.94	1.84	0.95	1.95	0.62	1.21	0.75	1.67	1.31	2.95	1.39	2.28	1.62	2.34	3.94	3.93
*Lake sediments (bulk fraction)*																
F_1_3	1.30	0.98	1.35	1.18	1.04	1.09	1.35	0.97	1.39	1.34	1.67	0.15	2.31	1.45	1.74	0.83
F_1_6	1.23	1.10	1.36	1.29	0.96	1.05	1.25	1.26	1.28	1.29	1.45	0.16	2.23	1.47	1.28	0.81
F_1_12	1.23	0.97	1.33	1.17	1.15	1.10	1.35	1.14	1.38	1.18	1.22	0.13	2.10	1.45	1.15	0.73
a/R	1.32	1.17	1.41	1.32	1.13	1.07	1.55	1.29	1.86	1.32	2.20	0.18	2.26	1.44	1.66	0.83
R9	1.37	1.06	1.50	1.25	0.98	0.88	1.27	1.37	1.30	1.06	1.47	0.16	2.21	1.68	1.30	0.88
R12	1.32	0.94	1.48	1.27	0.96	0.90	1.25	1.05	1.54	1.08	1.55	0.17	2.18	1.85	1.28	0.90
R18	1.38	1.13	1.55	1.35	1.01	0.99	1.31	1.19	1.35	1.22	1.52	0.16	1.87	1.63	0.67	0.76
T1	1.32	1.20	1.50	1.39	1.04	1.00	1.27	1.38	1.30	1.42	1.37	0.16	1.96	1.97	1.14	0.89
a/T	1.34	1.34	1.52	1.38	1.14	1.12	1.39	1.34	1.43	1.38	1.40	0.17	1.92	1.43	1.43	1.94
c/T	1.40	1.37	1.44	1.54	1.22	1.30	1.32	1.35	1.35	1.39	1.43	0.15	1.93	1.44	1.69	1.74
a/V	1.29	1.28	1.34	1.41	1.00	1.08	1.22	1.40	1.26	1.44	1.23	0.18	2.43	1.54	1.26	1.26
d/V	1.32	1.32	1.43	1.51	1.14	1.19	1.31	1.33	1.35	1.36	1.62	0.17	2.29	1.49	1.52	1.28
b/V	1.34	1.31	1.45	1.46	1.16	1.05	1.33	1.16	1.36	1.50	1.33	0.16	1.67	1.48	1.70	1.31
c/V	1.41	1.25	1.57	1.59	1.26	1.08	1.44	1.75	1.48	1.44	1.45	0.16	1.93	1.57	1.85	0.90
V3	1.31	1.83	1.40	2.05	1.05	1.47	1.28	2.05	1.32	2.10	1.49	0.20	2.19	2.25	1.48	1.05
*Fluvio-lacustrine sediments*																
EFF1	0.90	0.64	0.68	0.46	0.04	0.50	1.36	0.80	0.98	0.98	1.32	1.23	1.60	1.19	1.36	3.62
EFF2	0.88	0.63	0.83	0.56	0.03	0.46	1.37	0.80	0.72	0.71	1.26	1.17	1.18	0.88	1.09	2.90
EFF3	0.91	0.65	0.76	0.52	0.04	0.58	1.36	0.80	1.16	1.16	1.24	1.15	0.97	0.72	1.03	2.75
EFF4	0.89	0.64	0.78	0.53	0.04	0.48	1.26	0.74	0.89	0.89	1.18	1.10	1.29	0.96	1.32	3.50
EFF5	1.11	0.79	0.87	0.59	0.05	0.72	1.74	1.02	1.22	1.22	1.76	1.64	3.15	2.33	1.82	4.83
EFF6	1.13	0.81	1.34	0.91	0.04	0.56	1.42	0.83	0.89	0.88	1.48	1.38	1.29	0.95	1.11	2.95
EFF7	1.07	0.76	0.80	0.54	0.04	0.51	1.34	0.78	1.15	1.15	1.53	1.42	1.02	0.76	1.02	2.72
EFF8	0.98	0.70	1.19	0.81	0.04	0.47	1.35	0.79	1.29	1.29	2.54	2.37	1.47	1.09	1.37	3.65
EFF9	0.99	0.71	0.92	0.62	0.04	0.46	1.45	0.85	1.41	1.40	2.38	2.22	1.81	1.34	1.52	4.04
EFF10	1.06	0.75	0.78	0.53	0.04	0.46	1.45	0.85	0.95	0.95	1.75	1.64	1.88	1.39	1.25	3.32
EFF11	0.97	0.69	0.84	0.57	0.03	0.45	1.31	0.77	0.93	0.93	1.50	1.40	1.04	0.77	0.89	2.37
EFF12	0.72	0.52	0.78	0.53	0.03	0.39	1.17	0.68	0.76	0.76	1.22	1.14	1.06	0.78	0.74	1.96
EFF13	0.93	0.66	0.77	0.52	0.04	0.48	1.23	0.72	0.83	0.82	1.34	1.25	1.25	0.93	0.76	2.01
EFF14	1.00	0.71	0.76	0.51	0.05	0.60	1.42	0.83	0.88	0.87	1.64	1.53	2.48	1.84	1.35	3.59
*Meso-Cenozoic bedrock*																
EF1	1.02	0.73	1.00	0.67	1.87	1.10	2.18	1.27	1.87	1.87	0.42	0.39	0.44	0.33	1.17	3.11
EF5	0.98	0.71	1.00	0.68	1.06	0.62	0.82	0.48	0.56	0.56	0.95	0.89	0.89	0.66	1.23	3.27
EF6	0.65	0.47	0.86	0.58	0.64	0.38	0.63	0.36	0.86	0.86	0.65	0.60	0.89	0.66	1.07	2.86
EF16	1.15	0.82	1.19	0.80	0.97	0.57	1.16	0.67	1.49	1.48	1.23	1.15	1.35	1.00	0.93	2.47
EF22	1.03	0.74	0.65	0.44	0.89	0.52	0.57	0.33	0.59	0.59	1.11	1.04	1.46	1.08	1.48	3.93
EF26	1.10	0.79	1.10	0.75	0.81	0.47	0.72	0.42	0.74	0.74	1.03	0.96	1.18	0.87	1.09	2.89
EF28	0.88	0.63	0.92	0.62	2.15	1.26	0.84	0.49	0.52	0.52	0.91	0.85	0.89	0.66	1.08	2.86
EF30	0.76	0.55	0.85	0.57	1.52	0.89	0.85	0.49	0.87	0.87	0.92	0.85	0.72	0.54	0.54	1.44
EF34	1.06	0.76	1.01	0.69	1.14	0.67	1.11	0.65	0.57	0.57	1.07	1.00	0.85	0.63	0.89	2.37
EF57	1.16	0.84	1.10	0.75	0.71	0.42	1.28	0.75	1.42	1.42	1.20	1.12	1.27	0.94	1.11	2.96
EF59	1.04	0.74	1.25	0.84	0.83	0.49	1.33	0.78	0.77	0.76	0.96	0.90	0.86	0.64	0.80	2.12
EF76	1.00	0.72	0.90	0.61	1.52	0.89	1.97	1.15	3.18	3.17	0.65	0.61	0.80	0.59	0.90	2.40
EF77	1.07	0.77	0.76	0.52	0.69	0.40	1.67	0.97	3.10	3.09	0.78	0.72	0.68	0.50	1.08	2.86
EF78	1.06	0.76	1.00	0.67	0.63	0.37	0.82	0.48	0.84	0.84	1.16	1.08	1.28	0.95	1.05	2.80
EF79	1.44	1.03	1.89	1.28	2.77	1.63	3.32	1.93	3.42	3.40	1.61	1.50	1.01	0.75	2.67	7.09
EF80	1.26	0.90	1.13	0.76	1.16	0.68	0.90	0.52	0.92	0.92	1.04	0.97	1.01	0.75	2.60	6.91
EF81	1.02	0.73	1.00	0.68	0.79	0.47	0.88	0.51	0.68	0.68	0.85	0.80	0.36	0.27	0.71	1.88
EF82	1.04	0.75	1.15	0.78	1.00	0.59	0.91	0.53	0.71	0.70	0.97	0.91	0.98	0.72	0.74	1.95
EF83	1.67	1.20	2.28	1.54	4.50	2.64	5.00	2.91	7.72	7.69	2.90	2.71	2.54	1.89	8.04	21.3
EF84	1.56	1.12	2.14	1.45	2.17	1.27	3.75	2.19	2.89	2.88	1.82	1.69	1.15	0.85	3.01	8.01
EF85	0.45	0.32	0.62	0.42	0.47	0.27	0.91	0.53	0.70	0.70	0.44	0.41	0.51	0.38	0.73	1.94
EF86	1.46	1.05	1.23	0.83	1.18	0.69	1.08	0.63	0.83	0.83	0.94	0.88	0.49	0.37	0.87	2.30
EF87	1.12	0.81	1.10	0.74	1.13	0.66	0.88	0.51	2.03	2.02	0.93	0.87	0.58	0.43	1.27	3.37
EF88	1.14	0.82	1.33	0.90	1.65	0.97	2.91	1.70	1.50	1.49	1.24	1.16	0.36	0.26	0.94	2.49
EF89	1.33	0.95	1.46	0.99	1.77	1.04	1.83	1.07	0.47	0.47	1.77	1.65	0.47	0.35	1.47	3.91
EF90	0.85	0.61	0.95	0.64	1.60	0.94	2.77	1.61	2.14	2.13	0.94	0.88	0.56	0.42	1.11	2.96
EF91	1.57	1.13	1.86	1.26	2.10	1.23	3.06	1.78	3.15	3.14	1.58	1.47	1.25	0.92	3.28	8.73
*Quaternary deposits*																
EF39	1.00	0.72	1.08	0.73	1.22	0.72	1.14	0.67	0.98	0.98	0.08	0.22	1.65	1.22	1.38	3.66
EF41	0.95	0.68	0.92	0.62	1.09	0.64	1.11	0.65	0.92	0.92	0.08	0.21	2.91	2.16	1.29	3.44
EF49	1.43	1.03	1.33	0.90	1.35	0.79	1.85	1.08	1.50	1.50	0.11	0.30	3.57	2.64	1.88	4.99
EF50	0.87	0.62	0.58	0.39	0.99	0.58	0.75	0.44	0.44	0.44	0.12	0.33	1.86	1.38	2.05	5.46
EF51	1.17	0.84	1.23	0.83	0.62	0.36	1.16	0.67	0.62	0.61	0.06	0.15	1.40	1.04	0.96	2.56
EF52	0.66	0.48	0.71	0.48	0.40	0.24	0.67	0.39	0.40	0.40	0.07	0.20	1.51	1.12	1.25	3.33
EF54	1.27	0.91	1.07	0.72	2.03	1.19	1.62	0.94	2.11	2.10	0.29	0.78	4.71	3.49	4.90	13.01
EF60	0.89	0.64	0.91	0.62	0.96	0.56	1.11	0.64	1.03	1.02	0.05	0.13	1.17	0.87	0.80	2.13
EF61	1.24	0.89	1.16	0.79	0.65	0.38	1.01	0.59	1.09	1.09	0.08	0.22	1.29	0.96	1.36	3.62
EF62	1.16	0.84	1.17	0.80	1.26	0.74	0.94	0.55	1.20	1.20	0.11	0.29	2.38	1.77	1.81	4.80
EF63	0.91	0.65	0.98	0.66	0.88	0.52	0.86	0.50	0.88	0.88	0.06	0.15	1.00	0.74	0.97	2.57
EF65	0.94	0.68	0.97	0.66	1.22	0.72	0.95	0.55	0.98	0.97	0.12	0.32	1.45	1.07	1.98	5.27
EF67	1.02	0.73	1.00	0.68	1.03	0.60	1.00	0.58	1.03	1.02	0.08	0.20	1.22	0.90	1.28	3.42
EF71	1.04	0.75	0.91	0.62	0.97	0.57	0.75	0.44	1.03	1.03	0.08	0.21	1.58	1.17	1.29	3.43
EF74	1.51	1.09	1.43	0.97	1.40	0.82	1.14	0.67	1.47	1.46	0.11	0.29	1.86	1.38	1.84	4.88

The calculation of the average composition of local bedrock was based on the average composition of the lithoid samples of the outcropping sampled formations (Table 5). The composition was weighted relatively of the areal extension of each sampled lithoid formation in the analyzed area. In this way, the average composition of the local bedrock of the area of interest has been calculated and, based on this, the enrichment factors were performed. No significant differences between UCC and local bedrock values has been observed; however, the local bedrock composition represent a more coherent and useful data for the trace elements behavior and their enrichment factors calculation.

**Table 5:** Global UCC (McLennan et al. 2006) and local bedrock average composition (major oxides and trace elements)

	SiO_2_	Al2O_3_	Fe_2_O_3_	CaO	V	Pb	Cu	Zn	As	Co	Ni	Cr
	%	%	%	%	ppm	ppm	ppm	ppm	ppm	ppm	ppm	ppm
UCC (McLennan et al. 2006)	65.8	15.2	4.5	4.2	107	17	25	71	2	17	44	83
Local bedrock	54.2	14.6	5.5	6.7	108.1	17.6	35.0	93.3	5.6	14.3	36.1	78.9

### Enrichment factors based on global UCC composition

In the bulk fraction of the 15 lacustrine cores samples, As is always moderately enriched while Zn is slightly enriched in the a/R sample only.

As is enriched also in the clay fraction of lacustrine samples with a median of 2.93 and a maximum value of 5.16 in the sample a/T. Cu, Zn, and Pb are enriched in the same unique sample (V3).

In the 14 fluvio-lacustrine samples, As is moderately enriched in almost all samples (13 out of 14). Pb and Zn are enriched in very few specimens (1 and 2 respectively).

In the 27 Meso-Cenozoic bedrock samples, As is enriched in almost all samples (24) with a median of 4.5 and an extremely significant EF value of 21.36 in the sample EF85 which belongs to the Scisti silicei formation. Cu is also enriched in eight samples with a significant maximum value of 7.69 in the Gorgoglione Flysch sample EF83. Ni shows enrichment in two samples while Zn and Co are enriched in just one the sample EF83 (Gorgoglione Flysch).

In the 15 Quaternary deposit samples, As shows enrichment in all samples with a significant maximum of 1.01 in the sample EF54. Cu and Pb are slightly enriched respectively in one and three samples.

### Enrichment factors based on local bedrock composition

In the 15 bulk fraction of lacustrine samples, Pb is enriched in 9 samples while Zn is enriched in one sample.

In the 15 clay fraction of lacustrine samples, As is enriched in all the 15 sample while Zn is enriched in one sample.

In the 14 fluvio-lacustrine sediments, Zn and Pb are enriched in two samples.

In the 14 Quaternary fluvio-lacustrine samples, Co, Ni, and As are moderately enriched in one sample (EF54) while Pb is enriched in 4 samples.

## Discussion

### Lake sediment standard quality and the regulatory gap

The study of heavy metal contamination in an aquatic environment has attracted increasing attention because of its abundance, persistence, and environmental toxicity (Sun et al. 2016; Patel et al. 2018; Wang et al. 2019; Varol et al. 2020). It has been observed that to define more accurately the quality of sediments potentially affected by heavy metals and pollutants, a correct option could be an intensive comparison of applied sediment quality guidelines (SQGs) (Burton 2018; Birch 2018; Liu et al. 2019; Jones et al. 2019). Comparative evaluations of different SQGs for contaminants, in order to obtain an environmental quality and risk assessment of internal waters, has been intensively provided in recent years (Ali et al. 2016; Baran et al. 2016; Bhateria and Jain 2016; Gopal et al. 2017; Strady et al. 2017; Xu et al. 2017; Shyleshchandran et al. 2018; Zhao et al. 2018; Aung et al. 2019; Kulbat and Sokołowska 2019; Christophoridis et al. 2020). It has to be noted that SQGs for the assessment of sediment quality are very different across the different countries. The definition and the approaches to determine guidelines depends from crude percentile rankings of total concentrations of single chemical element to theoretical approaches depending on the biological available effect levels (Burton 2018). A large number of empirical water SQGs, based on matching chemical and biological-effects data, are well established and widely used. Empirical SQGs provide a useful screening tool to identify locations and chemicals of most concern, but are not regulatory criteria. In summary, SGQs vary from empirical to theoretical and are all characterized by a number of uncertainties and, as a consequence, it has been observed that a large number of reviewed SQGs show a considerable range in upper and lower guideline values (Birch 2018). Therefore, since a unique and homogeneous SQG value does not exist, there is a significant regulatory gap about dangerous substances in sediments of internal waters. The European Directive on Environmental Quality Standards (Directive 2008/105/EC), based on the Water Framework Directive (2000/60/EC), sets environmental quality standards (EQS) for the substances in surface waters (fluvial, lacustrine, transitional, and coastal) but not in its sediments. To overcome this gap, Member States may establish an approach for the definition of the admitted threshold values for dangerous substances in sediments. Commonly, the threshold values for dangerous elements in soils are used. Italy implemented the Water Framework Directive by a Legislative Decree “Decreto Legislativo 3 aprile 2006, n. 152, Norme in materia ambientale” which shows a great interest in aquatic systems but does not contain specific quality standards for river or lake sediments. Therefore, even in Italy, the threshold concentration values (CSC) for soils, laid down in the Single Environmental Text and subsequent amendments (Column A, Table 1, Annex 5, Title V, Part IV of Legislative Decree 152/06), are used as standards for sediment quality. In order to obtain a clear picture of the quality and dangerousness of the analyzed sediments, a comparison between samples analysis with some legislative references concerning the quality of the sediments has been performed. The following laws have been taken into consideration for the characterization of the chemical status of the analyzed sediments: Decreto 6 novembre 2003, n. 367 (D.M. 367/2003) “Regolamento concernente la fissazione di standard di qualità nell’ambiente acquatico per le sostanze pericolose” and Canadian Council of Ministers of the Environment (CCME 2011), “Canadian Sediment Quality Guidelines for the Protection of Aquatic Life”. These laws cover the limit concentrations of chemical elements in the marine environment including lake sediments.

Relatively of Italian legislation (D.M. 367/2003), in the bulk fraction of fifteen lacustrine samples, Cr exceeds the limit threshold in all samples, Ni in thirteen samples while Pb exceeds the limit just in one sample. Concerning the clay fraction of the same samples, Pb, Ni, and Cr overcome the limits in all samples (fifteen). In the fourteen fluvio-lacustrine samples, Ni and Cr exceed the limit threshold in all samples while Pb shows the same trend in three samples. In the twenty-seven Meso-Cenozoic lithoid samples, Ni exceeds limit values in twenty samples, Cr in all twenty-seven samples, and As in one sample. In the fifteen incoherent Quaternary parent rock samples, Ni and Cr exceed limit threshold in fourteen samples, Pb in eight samples, and As in three samples.

In the bulk fraction of fifteen lacustrine samples, almost all samples show higher values that exceed the limit threshold for Cu (thirteen samples), As (four samples), and Cr (fifteen samples) while Zn exceeds the limit in two samples. Also in the clay fraction of the same samples, almost all samples show higher values which exceed the limit threshold for some elements: Cu, Zn, and Cr exceed limits in all the samples, As in fourteen samples, and Pb in eleven samples. In the fourteen fluvio-lacustrine samples Cr exceeds limit values in all samples (fourteen), Zn in twelve samples, and As in ten samples. In the twenty-seven Meso-Cenozoic lithoid samples, Cr always exceeds limit values in all samples, Cu in twelve samples, As in eight samples, and Zn in two samples. In the fifteen Quaternary incoherent parent rock samples, Cr exceeds the limit threshold in all the samples and Cu and As in twelve samples while Pb and Zn exceed limit values in five samples.

Comparing the different concentration limits, a difference for chemical elements limits was observed (Table 6). It is important to note that the two considered regulations do not consider the same chemical elements. The Canadian ISQGs do not include V, Co, and Ni while the Italian D.M.367/03 does not consider V, Cu, Zn, and Co. Therefore, mismatches between the two regulations have been observed as well. For this reason, a comparison between chemical elements considered in both regulations (Cr, Pb, and As) has been proposed. Furthermore, limit values for Cu, Zn, and Ni have been observed as well.

**Table 6:** Threshold limit concentration for both Italian D.M. 367/03 and Canadian ISQGs 2001 regulations

	V	Pb	Cu	Zn	As	Co	Ni	Cr
	ppm	ppm	ppm	ppm	ppm	ppm	ppm	ppm
Italian D.M. 367/03	nd	30	nd	nd	12	nd	30	50
Canadian ISQGs 2001	nd	35	35.7	123	5.9	nd	nd	37.3

nd = not detected.

In the lacustrine sediments (both bulk and clay fraction), Cr exceeds the limit in all samples for Italian D.M. 367/03 and Canadian ISQGs. In the fourteen fluvio-lacustrine samples, for ISQGs, Cr exceeds limit values in all samples, while for D.M. 367/03, Cr overcomes the limit in fourteen samples. In the Meso-Cenozoic sediments, for ISQGs regulation, all samples exceed the limits, while for D.M. 367/03, the threshold is exceeded in twenty-five samples. All the fifteen quaternary samples show values exceeding the limit values for Canadian ISQGs and for D.M. 367/03 exceeds limit threshold in almost all samples (fourteen out of fifteen).

In the bulk fraction of the fifteen lacustrine samples, Pb overcomes the limit threshold just in one sample relatively to Italian D.M. 367/03. Differently, in the clay fraction, Pb overcomes the limits in all samples (for D.M. 367/03) and in eleven samples (for ISQGs). In the fluvio-lacustrine sediments, Pb overcomes the limit threshold in two and three samples, for Canadian ISQG and D.M. 367/03 regulations, respectively. No samples exceed Pb limit values in the twenty-seven Meso-Cenozoic sediment samples. In the fifteen quaternary samples, for Canadian ISQGs, Pb exceeds the limit threshold in five samples, while for D.M. 367/03, Pb overcomes limit threshold in eight samples.

For the fifteen bottom lake sediments (clay fraction), for the Canadian ISQG, fourteen samples exceed the threshold, while for the Italian D.M. 367/03, no sample exceeds the limit threshold. For ISQGs, As in the fourteen fluvio-lacustrine sediments exceeds the limit in ten samples, and for D.M. 367/03, no samples exceed limits. In the twenty-seven Meso-Cenozoic sediments, As exceeds the limit in eight samples for ISQGs while just one sample exceeds limit relatively to D.M. 367/03. Finally, in the fifteen incoherent Quaternary deposit samples, As exceeds the threshold in twelve samples, for ISQGs, and three samples for D.M.367/03.

Concerning the other trace elements, relatively to Canadian ISQGs regulation, Cu exceeds limit threshold in a large number of samples. In fifteen lacustrine sediments, Cu overcomes the limit in thirteen and fifteen samples, in the bulk and clay fraction, respectively. In the fourteen fluvio-lacustrine samples, Cu exceeds the limit threshold in twelve samples. Always, twelve samples exceeds Cu limit values in the twenty-seven Meso-Cenozoic lithoid samples, while in the quaternary deposits samples, Cu overcomes the limit threshold in almost all samples (twelve out of fifteen).

For Canadian ISQGs, Zn exceeds limit values in all fifteen lacustrine samples (clay fraction), while in the fluvio-lacustrine samples, the Zn limit threshold is exceeded in twelve samples out of fifteen.

The values of Ni, relatively to Italian D.M. 367/03, overcome the limit values in a large number of samples. Ni in all lacustrine (clay fraction), fluvio-lacustrine, and quaternary sediments samples exceeds the limit threshold.

In the bulk fraction of the fifteen lacustrine samples, Ni overcomes limit the threshold in thirteen samples while, in the Meso-Cenozoic lithoid samples, Ni exceeds limit values in twenty samples out of twenty-seven.

**Fig. 7:**
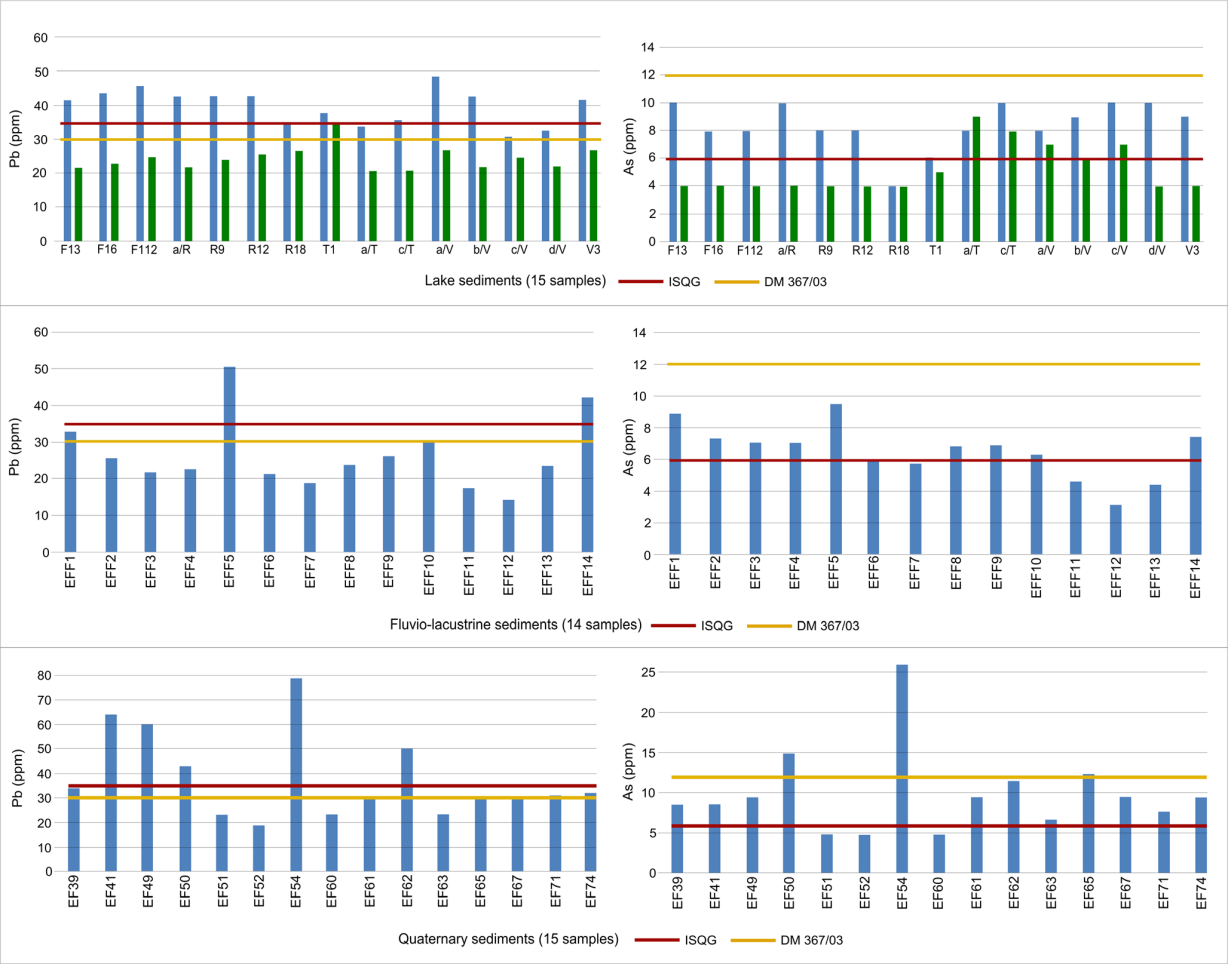
Chemical composition of selected trace elements (Pb and As) in relation with threshold values of the considered regulations (Canadian ISQGs and Italian D.M. 367/03). Note that, in lacustrine sediments, blue lines represent clay fraction while green line represent bulk fraction

Figure 7 shows selected trace element (Pb and As) composition in the analyzed samples, in relation with the considered normative admitted limit threesholds.

In order to establish the quality of an environment from the environmental and pollution point of view, it would be useful to define a common and homogeneous regulatory reference for all countries, taking into account the same pollutants in the different environments. Considering the regulatory gap affecting fluvio-lacustrine and lacustrine sediments, we will discuss the data of the Pietra del Pertusillo catchment mostly considering the distribution of elements with respect to the local upper continental crust. This may represent an innovative approach under similar conditions worldwide.

### Minero-chemical processes and trace elements within the reservoir sediments

Lacustrine sediments are commonly used for the study of the erosion history of the watershed and to understand the processes, both natural and anthropogenic, affecting the concentration and the mobility of trace elements in water and sediments (Islam et al. 2015; Gao et al. 2017).

Mineralogy of studied samples reflects the parent rock composition. Quartz is the main allogenic phase, derived from the silico-clastic rocks outcropping around the lake basin. Only in the samples from the cores T and V, calcite is higher than quartz, a finding consistent with the higher values of total organic carbon (TOC) in these cores. Dolomite, likely supplied by the Campano-Lucana carbonate platform units, was found only in the F core. The distribution of feldspars in lacustrine sediments, as well as quartz, also reflects the composition of lithologies outcropping along the drainage basin. Higher amounts of feldspars were found in the cores R, T, and V near the silico-clastic deposits (Gorgoglione and Albidona fms.). Clay minerals like illite and chlorite represent the weathering product of rocks in the drainage basin. The presence of chlorite could be associated with the weathering of mafic silicates-rich rocks, such as Lagonegro basin lithologies (Scisti silicei and Galestri fms.), Liguride unit deposits, and Albidona Fm.

The elemental concentration of sediments not only depends on anthropogenic and lithogenic sources but also upon pH, organic matter content, mineralogical composition, and depositional environments of sediments (e.g., Alyazichi et al. 2014, 2016; Hsu et al. 2016). The dissolved concentrations of trace metals with depth are expected to be controlled by sorption processes and/or by the dissolution/precipitation of their discrete secondary minerals and organic matter (Ho et al. 2012; Alyazichi et al. 2016). Clay and organic matter contents are often used to calculate ‘corrected’ background values for trace metal concentrations in soils and sediments. Clay minerals largely affect metal clay retention (e.g., Mongelli 1995); Malla (2002) and Jones et al. (2019), for instance, observed the effective adsorbing of 2 : 1 clay minerals of bivalent cations such as Cu, Pb, Cd, and Zn. The solubility of trace elements, such as Cu, Cr, Pb, and Zn (Alyazichi et al. 2015), may be also associated to Mn oxides through coprecipitation and substitution and may increase when Mn is reduced (Negra et al. 2005).

In the studied samples, the trace elements which could be relevant from an environmental point of view are Cr, Cu, Zn, As, and Pb. Determination of toxic metals in water sediments makes it possible to assess the potential migration of metals from sediments to water and the potential toxicity of metals (Pejman et al. 2015; Palleiro et al. 2016).

Cr is a very common pollutant in soils, aquifers, and superficial water, where it is stable in hexavalent form (Margiotta et al. 2012; Margiotta et al. 2014). Chromium occurs in more than one oxidation state, and its solubility in the soil depends on pH and mineral content. Cr^3+^ has low solubility, whereas hexavalent chromium is adsorbed onto Fe, Mn, and Al oxyhydroxides and clay minerals (e.g., Davis and Lackie 1980). Recently (Mwamburi 2016) and less recent works (Griffin et al. 1977) demonstrated that the adsorption of Cr by clay minerals was found to be highly dependent upon the physical-chemical properties of the clay minerals. This is consistent with the presence of Cr in the analyzed samples where Cr is enriched in the fine fraction of lake sediments and, more in general, Cr is more abundant in samples with larger occurrence of clay minerals such as chlorite.

Yang and Rose (2005) suggested that Cu occurrence in soils mostly depends on mineral composition, and Cavallaro and McBride (1978) observed that clayey minerals act as the receptor for Cu in non-calcareous soils. Cu, in our case, is particularly abundant in rocks containing mafic minerals (Gorgoglione, Galestri, and Albidona Fms) and is enriched in some present-day sediments at Maglia river mouth, suggesting thatCu is associated with the secondary phases deriving by the weathering of mafic silicates.

Zinc is adsorbed by clay minerals (Uddin 2017), carbonates, or oxides-hydrates, and Tessier et al. (1980), Kuo et al. (1983), and Hickey and Kittrick (1984) found that the highest percentage of Zn in sediments is associated with Fe-Mn oxides. Zn is enriched with respect to the local upper continental crust in very few samples including one sample from the core R and two fluvio-lacustrine samples showing EF > 2, suggesting that naturally occurring local conditions may affect the Zn distribution in the analyzed catchment.

Pb, which is enriched in most of the lacustrine sediments and specifically in the fine fraction, is generally effectively adsorbed onto clay minerals (up to 95% of available Pb) at pH ranging from 3.0 to 4.5 (Scrudato and Estes 1975). Due to its great mobility, Pb can be mobilized and transported from the outcropping rocks toward the reservoir, where it is likely absorbed by the most reactive phases, including clay mineral and Fe-Mn oxyhydroxides.

As is constantly enriched in the lacustrine samples, and it is well-known that the occurrence of elevated concentrations of As in sediments can compromise soil and water quality since it is one of the most toxic elements in the environment. The toxicity and mobility of arsenic in the environment are determined by a complex series of controls dependent on mineralogy, chemical speciation, and biological processes (Bowell et al. 2014). As occurs as multiple oxidation state element, and its distribution in sediment depends on the sediment redox state (Yang and Rose 2005). In soil and sediments, the availability of As is also affected by the chemical and physical features of sediments including the presence of clay minerals, Al-Fe hydroxides, organic matter content, pH, Eh, and cation-exchange capability (El Bilali et al. 2002; Shrivastava et al. 2015). The mobilization of As into the hydrosphere is linked with a combination of natural processes such as weathering reactions, biological activity, mineral-water interactions, and through a number of other anthropogenic activities (Chakraborti et al. 2017). As-enriched sediments are a possible source of As to water (Ying et al. 2013) and, in our reservoir system, this element is largely concentrated in the fine fraction of the lacustrine samples (EF > 2 with respect to the local upper crustal composition). This finding reflects the adsorption capability of the fine fraction of the sediment from which, potentially, significant amount of As may move toward the fluid matrix causing water pollution.

## Conclusions

Freshwater reservoir role a great interest for the evaluation of environmental contamination as they are generally located close to several human activities and sources of pollution. In particular, lake sediments act as good receptors for different pollutants; therefore, this kind of sediments has a great impact on the health of citizens and environment. For these reasons, and for the first time, the Pietra del Pertusillo reservoir lake sediments and fluvio-lacustrine sediments have been analyzed to assess environmental quality. Although the definition of the pollution status of a natural environment is an issue of great importance worldwide, in Italy, specific regulatory values for the element threshold concentration for the lake and river sediments do not exist. For this reason, soil threshold values are used as a standard for sediments of internal waters. Further, in order to define the qualitative state of the reservoir, by comparing elemental concentrations of analyzed sediments with limit values of the considered regulations (Italian D.M. 367/03 and the Canadian ISQGs), significant differences and mismatches were observed.

Within this scenario, the evaluation of the environmental quality depicted by the lacustrine and fluvio-lacustrine sediments of the Pietra el Pertusillo reservoir has been performed on the basis of the enrichment factors with respect to the average composition of a restored local upper continental crust. We suggest this method as an innovative standard in similar conditions worldwide. In our catchment, the enrichment factors indicate that the trace elements that may be of some environmental concern are Cr, Cu, Zn, As, and Pb, although the distribution of these elements, at this stage, appear to be mostly driven by geogenic processes. However, attention has to be paid to As, constantly enriched in the lacustrine samples and especially in the fine-grained fraction. Large concentrations of As, considered one of the most relevant contaminant for the environment and health, may potentially compromise sediments and water quality. Arsenic is highly toxic in its inorganic form, and soluble inorganic arsenic can have immediate toxic effects for human health.

Finally, the effects of toxic pollutants on health and environment do not change for the different countries of the planet and therefore this criticality leads us to suggest a worldwide and homogeneous regulatory framework for an effective assessment of the quality status of natural materials in different environments.

## Data Availability

All data generated or analyzed during this study are included in this published article (and its supplementary information files).
